# Assessing Performance and Safety of Feeding a Standardized *Macleaya cordata* Extract to Calves

**DOI:** 10.3390/ani12202875

**Published:** 2022-10-21

**Authors:** Ray A. Matulka, Janaka Wickramasinghe, Juliane Dohms, Flavio Rodrigues Borges Ribeiro, Ranga Appuhamy

**Affiliations:** 1 Burdock Group, 859 Outer Road, Orlando, FL 32814, USA; 2 Department of Animal Science, Iowa State University, 313G Kildee Hall, Ames, IA 50011, USA; 3 Phytobiotics, Futterzusatzstoffe GmbH, Wallufer Str. 10a, D-65343 Eltville, Germany

**Keywords:** cattle, feed, sanguinarine, milk replacer, residue, safety, hematology, serum chemistry, *Macleaya cordata*

## Abstract

**Simple Summary:**

A preparation of the *Macleaya cordata* extract (Sangrovit^®^) provides a unifying taste to feed for cattle. However, information is needed to see if the effect occurs when fed in milk replacer or starter feed to calves and residues of the extract end up in the tissues. Male and female calves (~5 d of age; 5/sex/group) were provided Sangrovit^®^ in milk replacer and starter feed at control (0.0 Sangrovit^®^), 2.0 and 4.0 g (D1), 5.0 and 10.0 g (D2) and 10.0 and 20.0 g (D3) Sangrovit^®^/day, respectively, for 90 days. Body weight, feed intake and blood-derived endpoints were evaluated along with tissue residue levels of Sangrovit^®^. Compared to the control group, Sangrovit^®^ tended to increase milk-replacer intake (4%). Average daily gain did not change, and some blood chemistry and hematology parameters changed with Sangrovit^®^ intake, but were within the ranges of healthy calves. Residues of Sangrovit^®^ were located in the edible tissues of the calves. Sangrovit^®^ can be added to milk replacer and calf starter feeds with no adverse effects on feed intake, health, or growth of the calves.

**Abstract:**

This study examined the effects of Sangrovit^®^, a *Macleaya cordata* plant extract (MCE) preparation on feed intake, growth, blood chemistry, and tissue-residue levels of calves. Twenty male and 20 female calves (~5 d of age) were assigned to one of four daily Sangrovit^®^ doses: 0.0 and 0.0 (CTL), 2.0 and 4.0 (D1), 5.0 and 10.0 (D2), and 10.0 and 20.0 (D3) g/calf in pre-weaning (5 to 49 d of age) and post-weaning (50 to 95 d of age) periods, respectively. Sangrovit^®^ doses were fed in milk replacer pre-weaning and top-dressed on calf starter post-weaning. Milk replacer and calf starter intake was recorded daily. Body weight, hematology, and serum chemistry were measured at 5, 49, and 95 d of age. Calves were slaughtered at 95 d of age for MCE tissue residue analysis. Compared to CTL, D1 increased milk-replacer intake (4.90 to 5.09 L/day), but decreased calf starter intake pre- (0.65 to 0.53 kg/d) and post-weaning (3.42 to 3.20 kg/d). No Sangrovit^®^ dose affected average daily gain. The hematology and blood chemistry of all treatment groups fell within the ranges of healthy calves. Results showed no adverse effects of MCE on health and growth performance of calves when fed up to 10.0 g/calf/day pre-weaning and up to 20.0 g/calf/day post-weaning.

## 1. Introduction

*Macleaya cordata* (commonly called Plume Poppy) is a plant of the family Papaveraceae, native to eastern Asia and grown throughout Europe as ornamental plant. *M. cordata* is a hollow-stemmed, leafy, fast growing deciduous perennial plant that can grow to 2.0 m high by 1.0 m wide [1]. *M. cordata* aerial portions contains isoquinoline alkaloids at approximately 10.85 mg/g dry plant material [2], mainly sanguinarine and chelerythrine that are bitter in taste, with potential use in animal feed as the bitter taste at low levels may not register as “bitter” in the animal, but only mask other variable flavors that could transiently decrease feed intake [3,4].

Sangrovit^®^ (Phytobiotics, Eltville, Germany) is a feed additive made from a *M. cordata* extract (MCE, 3.5%), which contains a minimum of 1.5% sanguinarine. Kantas et al. [5] demonstrated significantly increased feed consumption in weaning pigs fed diets that included MCE. Feeds containing MCE have also been evaluated as an appetizing agent in poultry- and fish-farming studies [6,7,8]. Studies have shown cattle to prefer a bitter taste as opposed to sweet taste, when sweet, sour, bitter, and salty tastes were examined [9]. Research using ruminant animals has demonstrated that Sangrovit^®^ improves the gain-to-feed ratio of sheep [10] and crossbred Nellore–Angus bulls [11]. Moreover, calves increased starter intake when flavored with the same flavoring ingredient as was included in the milk replacer fed to the calves [12]. Therefore, the addition of a flavoring agent such as Sangrovit^®^ that contains MCE to both the milk replacer and starter calf feed has the potential to enhance overall feed intake in young cattle. However, few studies have been performed to examine the safety of MCE consumption in young calves on milk and transitioning to solid feed. Additional analyses for potential MCE residues remaining in typically consumed organs and tissues would enhance the overall safety information for consumers on MCE as a flavor ingredient in commercial livestock. In this study, the objectives were to determine the consumption by calves of a standardized preparation of MCE for 90 days when transitioning from milk replacer to solid feed and to evaluate the residue levels of sanguinarine and chelerythrine in selected organs and tissues of the calves at the end of the study.

## 2. Materials and Methods

### 2.1. Test Substance

The experiments were conducted using two manufactured preparations of MCE, (trade name Sangrovit^®^) as the test substance. Each Sangrovit^®^ preparation was standardized to contain 3.5% MCE (providing at least 1.5% sanguinarine). The remaining content of Sangrovit^®^ is composed of ingredients approved for feed use. The level of sanguinarine and chelerythrine in Sangrovit^®^ was measured before adding it to milk replacer or calf starter feed. The water-soluble form of the MCE preparation (Sangrovit^®^ Stat Pak) and the dry-mix form of the MCE preparation (Sangrovit^®^ G Premix) were analyzed and found to contain 0.82% and 0.39% sanguinarine and 0.33% and 0.15% chelerythrine, respectively. The test ingredients were provided by Phytobiotics Futterzusatzstoffe GmbH (Eltville, Germany).

### 2.2. Experimental Animals, Housing Conditions and Diets

The trial utilized forty (20 male and 20 female) crossbred (Holstein × Angus or Angus–Simmental) calves that were 5 ± 2 days of age at the study’s start with an initial live weight of 45.2 kg. The study was conducted at the Dairy Research and Teaching Unit at Iowa State University (ISU-Dairy) from September to December 2019 (Ames, IA, USA). The calves confirmed to be negative for bovine viral diarrhea [13] were purchased from a commercial farm (Milk Unlimited, Atlantic, IA, USA). All calves satisfied study inclusion criteria, such as <10 d of age, birth weight > 30 kg, and satisfactory health status. The calves were housed individually in calf hutches (2.4 m × 1.4 m × 1.3 m) bedded with straw throughout the study. The space allocated was 3.4 m^2^/calf, which is the usual recommendation for a 2- to 4-month-old dairy calf in the US. The condition of bedding in hutches was checked daily and new bedding was added every other day, facilitating animal comfort. The calves were housed outside under ambient temperature of the fall to winter season (September to December). The calves were housed outside under natural light, and an adjacent light source was adequate to observe animals during the night hours.

The Sangrovit^®^ Stat Pak was provided in milk replacer (Land O’Lakes^®^, St. Louis, MO, USA) and the Sangrovit^®^ G premix was top-dressed onto a commercial calf starter ration (Farmers Win Coop^®^, Houston, MN, USA), which met the nutrient requirements of calves. The ingredient and nutrient composition of the milk replacer and starter ration are provided in Table 1. The milk replacer did not contain mannan-oligosaccharides, which are otherwise present in the regular milk replacer of the manufacturer. It had a flavoring agent at 0.04 g/kg, which was smaller compared to the inclusion rates of Sangrovit^®^ (0.28, 0.70, and 1.40 g/kg of dry milk replacer). The calf starter did not contain antibacterial, phytogenic, or appetite-enhancing additives. Confirmation of the Sangrovit^®^ marker components (sanguinarine and chelerythrine) was conducted for Sangrovit^®^ Stat Pak and Sangrovit^®^ G Premix, and the powdered milk replacer that contained the Sangrovit^®^ Stat Pak. Because Sangrovit^®^ G Premix was top-dressed onto the calf starter and thus not homogenously mixed into the daily feed portions, the starter feed was not analyzed for sanguinarine and chelerythrine concentrations.

### 2.3. Feed Analysis

Approximately 200.0 g of composite starter feed samples, each representing each batch of feed used throughout the study, was dried in an oven at 60 °C for 72 h to determine dry matter (DM) content. The dried samples were then analyzed (Cumberland Valley Analytical Services, Waynesboro, PA, USA) for crude protein (CP) (N × 6.25; AOAC, 2000; method 990.03), neutral detergent fiber (NDF) [14], acid detergent fiber (ADF) (AOAC, 2000; method 973.18), starch ([15]; with correction for free glucose), and ether extract [EE (AOAC, 2000; method 920.39)].

### 2.4. Study Design and Execution

The study design was a randomized complete block design, in which the calves were acclimated to new housing environment for two days, blocked by sex, and randomly assigned to the treatments. The treatments consisted of a control (CTL) and three increasing doses (D1, D2, and D3, respectively) of two Sangrovit^®^ products, Sangrovit^®^ Stat Pak and Sangrovit^®^ G premix administrated during pre-weaning (5 to 49 d of age) and post-weaning (50 to 95 d of age) stages, respectively. The corresponding doses (Table 2) were pre-weighed into plastic bags and stored in a refrigerator until use.

The experimental unit was the individual calf, and each treatment was assigned to 10 calves (*n* = 10). The average age of calves in each treatment group was similar at 5 days. The 40 hutches were arranged in four rows in the experimental location, and each hutch row represented calves from all four treatments. Individual calves had free access to clean drinking water and a starter feed offered in two separate buckets throughout the study. Each bucket held a maximum of 8.0 kg of water or starter feed. Water and starter feed levels in the buckets were checked and refilled, if necessary, twice per day (morning and afternoon) throughout the study. The weights of liquid milk replacer offered to and left over by individual calves were recorded daily until calves were completely weaned. Additionally, 50.0 mL sample of liquid milk replacer prepared for each treatment was collected and saved in −20 °C once in every 5 days throughout this period.

During the pre-weaning period, one half of each dose (Table 2) was mixed separately with 3.8 kg of dry milk replacer at each feeding. A 200.0 g sample from each daily batch of dry milk replacer containing Sangrovit^®^ was saved under refrigerated conditions until being shipped for sanguinarine and chelerythrine concentration analysis. The remaining dry mixture (3.6 kg) was reconstituted with 27 kg of warm water (38 °C) to prepare liquid milk replacer adequate to feed 10 calves per treatment at each feeding (3.0 kg of liquid milk replacer per calf per feeding). Individual calves were bottle-fed with 6.0 kg of milk replacer (12% solid) at two feedings (3.0 kg per feeding at 0700 and 1800 h) until 42 days of age. In addition to Sangrovit^®^ doses, a coccidiostat containing decoquinate (Deccox-M^®^, Zoetis, Parsippany-Troy Hills, NJ, USA) was fed in the milk replacer to each calf (2.0 g/day) until 28 d of age.

Starting at 43 days of age, all the calves were partially weaned by eliminating the morning milk feeding and received only 3.0 kg/day milk replacer until they were weaned completely at 49 days of age. During the partial weaning period, full daily dose mixed in 3.6 kg of dry milk replacer was reconstituted with 27 kg of warm water and offered to each group of 10 calves. The weights of liquid milk replacer offered to and left over by individual calves were recorded daily until calves were weaned completely. Additionally, 50 mL sample of liquid milk replacer prepared for each treatment was collected and saved in −20 °C once in every 5 days throughout the suckling period. The dry samples were composited within each week on site and shipped along with the liquid milk-replacer samples for sanguinarine and chelerythrine concentration analysis (ATC Scientific, North Little Rock, AR, USA). Once the calves were weaned, the Sangrovit doses (Sangrovit^®^ G premix, Table 1) were fed with the starter feed.

During the post-weaning period, 110% of the previous day’s intake of starter feed was measured into the feed bucket in the morning, and the daily dose of Sangrovit^®^ G premix (Table 1) was top-dressed and offered to each calf. To minimize potential losses to the air, the top-dressed dose was mixed with the topmost layer (about 1–2 inch deep) of starter feed in each bucket before calf feeding. When needed, the starter feed buckets were refilled in the afternoon by placing the old feed mixed with Sangrovit^®^ on top of the new feed to facilitate complete consumption of Sangrovit^®^ dose and availability of Sangrovit^®^ over the 24 h period. The amounts of starter feed offered to and leftover by individual animals were recorded daily throughout the study.

The general health status of the calves and the housing were inspected twice a day. The feed intake, health/illness, and adverse events were analyzed daily and carefully recorded during the whole period of experiment (90 days). Individual live weights (BW) of the calves were recorded at the beginning, mid-point (approximately 45 days), and at the end of the study. The feed-conversion ratio (gain:feed ratio) in each period was calculated by dividing the average daily gain by the corresponding average feed intake (kg/d).

### 2.5. Study Parameters

#### 2.5.1. Body Weight

Body weights of individual calves were recorded on day 0, 45, and 90 of the study corresponding to 5, 50, and 95 days of age. Body weight was measured using a digital scale (0.1 kg resolution) with a metal cage bolted onto it. The scale was capable of weighing accurately, even if animals leaned against the cage.

#### 2.5.2. Blood Collection and Analysis

Following body-weight measurements, on days 0, 45, and 90 of the study, blood was drawn from the jugular vein of individual animals after morning feeding into two 2.0 mL vacutainer tubes, one with sodium-citrate and the other with EDTA for prothrombin and fibrinogen analysis, and other hematology parameter analyses, respectively. An additional blood sample was collected into a 10.0 mL tube without anticoagulants for clinical chemistry analysis. At each bleeding, blood in EDTA tubes were stored under refrigerated conditions until they were shipped for the analysis. Blood in the other tubes were centrifuged at 2000× *g* at 4 °C for 15 min to separate serum and plasma, which were then stored at −20 °C until they were shipped for analysis. Samples stored in refrigerated and freezing conditions were shipped in the same conditions (e.g., with cool packs and dry ice, respectively) within two days post-bleeding using an overnight courier network for clinical pathology analyses.

All blood analyses were carried out under good laboratory practice (GLP) guidelines at Quality Vet Laboratory (Davis, CA, USA). This laboratory provides testing services for GLP-regulated (21CFR Part 58 compliant) trials in areas of hematology, blood chemistry, and blood coagulation.

#### 2.5.3. Clinical Chemistry and Hematology Parameters

Serum clinical chemistry analysis included total protein (TP) and nitrogen (N), alkaline phosphatase (ALP), alanine aminotransferase (ALT), aspartate aminotransferase (AST), lactate dehydrogenase (LDH), γ-glutamyltransferase (GGT), creatinine (CREAT) and creatine kinase (CK), amylase (AMY), cholesterol (CHOL), glucose (GLU), total bilirubin (TBIL), blood urea nitrogen (BUN), and the minerals calcium (Ca), inorganic phosphorus (iP), phosphorus (PHOS; P), sodium (Na), potassium (K), chloride (Cl), and magnesium (Mg). Those analyses were carried out using an AU400e chemistry analyzer (Beckman Coulter, Brea, CA, USA).

Blood samples were processed to evaluate the following hematological parameters: Red (RBC) and white blood cell (WBC) counts, hemoglobin (HGB), platelets (PLT), prothrombin time (PT), fibrinogen, absolute basophils (BASO), eosinophils (EOS), lymphocytes (LYMPHS), monocytes (MONO) and neutrophils (NEUT), mean corpuscular hemoglobin (MCH; in pg and g/dL formats), mean corpuscular volume (MCV), and mean platelet volume (MPV). Blood samples for hematological analysis were analyzed with ADIVA 120 system (Siemens Healthneers, Erlangen, Germany) and STA Compact Max^®^ coagulation analysis system (Diagnostic Stago Inc., Parsippany, NJ, USA).

#### 2.5.4. In-Life Observations

The water levels in the buckets were monitored two to three times per day to make certain that animals received enough drinking water. When milk replacer was being fed, individual animals were observed and helped (e.g., for locating the bottle at the beginning) to confirm that animals consumed the milk replacer allowance at each feeding. If milk-volume intake decreased less than 50% for more than two consecutive days, those animals were checked and treated (if necessary) by a field veterinarian. Observations and treatments of sick animals were recorded systematically. The diarrhea scores were recorded daily on a scale of 1 to 4 (1 = normal, 2 = viscous feces, 3 = runny feces, and 4 = runny feces with splatters) until calves were 28 days of age. No calf had a fecal score of 2.0 or greater and required electrolyte supplementation during the study. One calf in the control group became bloated (ruminal tympany) during the pre-weaning period, such that a veterinarian was requested and physically relieved the gas (abomasal bloat) by use of a catheter. No other adverse effects were reported during the in-life phase of the study.

#### 2.5.5. Necropsy Procedures

Calves completing 90 days of treatments were matched for age and treatment and assigned to four groups (10 calves per group) that were slaughtered on four separate days. The age at slaughter was maintained similar (95 ± 2 days) across the treatment groups. About 2 h after morning feeding (0900 h) with control or treatment diets on the day of necropsy, calves were transported to the livestock infectious-disease isolation facility (LIDIF) at Iowa State University (Ames, IA, USA) for slaughter. A licensed field veterinarian at Iowa State University performed slaughter using a penetrating captive bolt pistol followed by immediate exsanguination. The same veterinarian helped in opening the body cavities for tissue collection. Tissue samples weighing approximately 50 g of liver (a cross section of each lobe), kidney (from each kidney), muscle (loin, flank, and hind leg, separately), and fat (subcutaneous, mesenteric, and perirenal, separately) were collected. Calves in the control group were euthanized and tissues were collected first to minimize potential contamination. All samples were snap frozen in liquid nitrogen and stored at −80 °C until being shipped on dry ice to the ATC Scientific laboratory (North Little Rock, AR, USA) for sanguinarine and chelerythrine concentration analyses. The slaughter and sample collection from all 10 animals were completed within about 3 h on each day.

### 2.6. Measurement of Sanguinarine and Chelerythrine Residues

#### 2.6.1. Sample Preparation and Purification

Utilizing a standardized, validated process, the dry milk samples (control and treatment) were homogenized by grinding, then dissolved in a solution of acetonitrile:water (95:5), sonicated and then centrifuged. The sediment was extracted again with a solution of methanol:water (90:10), sonicated and centrifuged, with the two resulting supernatants combined. Magnesium sulfate was then added, and the solution defatted with hexane and agitation. After centrifugation, the hexane was removed, and the samples dried then reconstituted with methanol.

#### 2.6.2. Sample Analysis

The level of sanguinarine and chelerythrine in the samples were analyzed by ultra-high pressure liquid chromatography coupled to triple quadrupole mass spectrometry (UHPLC-MS/MS) equipped with Accucore aQ C18 Polar Endcapped 2.6 µm, 2.1 × 100 mm liquid chromatograph column. The mobile phase consisted of (A) water + 0.1% formic acid, and (B) acetonitrile + 0.1% formic acid. The signal was detected by an ESI positive ionization detector with MS/MS. For each matrix, a calibration curve was obtained using a matrix-matched standard of sanguinarine and chelerythrine (Sigma-Aldrich, St. Louis, MO, USA), prepared in a blank matrix (without MCE), for each organ/tissue.

#### 2.6.3. Analytical Method Validation

The measuring/recording instruments used were calibrated before use. The method for the detection and quantification of sanguinarine and chelerythrine concentrations were validated in the blank matrix before performing sample analysis. Measured amounts of standard were added to the blank matrix and then extracted to isolate sanguinarine/chelerythrine as described above. Three different concentrations of standard were added to each matrix, and measurement of three replicates (*n* = 3) was performed for each concentration. The blank matrix (control) was also extracted and analyzed. The parameters involved in the analysis were validated, including accuracy, detection limit, linearity, precision, quantification limit, range, and specificity. Recovery (%) of sanguinarine and chelerythrine in each matrix was calculated as the percentage of the detected level to the level that the standard was initially added.

### 2.7. Calculations and Statistical Analysis

Dry matter intake (DMI) during the pre-weaning period was calculated, adding together the dry matter from milk replacer and DM from starter feed. Average daily gain (ADG) was calculated for pre- and post-weaning periods by taking the difference between baseline and mid-point body weight, and mid-point and final body weight divided by corresponding age differences in days, respectively. The gain-to-feed ratio during pre- and post-weaning periods were calculated taking the ratio between the ADG and corresponding average DMI (ADG:DMI).

The treatment’s effects on starter intake, milk replacer intake, and total DMI during either pre- or post-weaning periods were analyzed using the repeated option in MIXED procedure [16] of SAS with the unstructured variance-covariance structure (SAS Institute Inc., Cary, NC, USA). The following mixed-effect model was applied.

Y_ijklmn_ = μ + T_i_ + W_j_ + S_k_ + B_l_ + (T × S)_ik_ + C_m_ + e_ijklmn_ (1)

where Y_ijklmn_ = the response variable of interest, μ = overall mean, T_i_ = fixed effect of treatment (i = CTL, D1, D2, and D3), W_j_ = fixed effect of age in weeks, S_k_ = fixed effect of sex, B_l_ = fixed effect of breed, (T × S)_ik_ = fixed interaction effect between treatment and sex, C_m_ = random effect of calf, and e_ijklmn_ = random residual error. The BW, ADG, ADG: DMI, blood chemistry and hematology were analyzed across periods by including period, and period by treatment interaction, in the model, excluding the week effect. The associations of Sangrovit^®^ dose with each response variable was tested using linear, quadratic, and cubic orthogonal contrasts. When blood chemistry and hematology were analyzed, the corresponding baseline measurement was used as a covariate.

## 3. Results and Discussion

### 3.1. Feed Intake and Growth

The effects of Sangrovit^®^ dose on feed intake and growth performance of calves during the pre-weaning (until 49 days of age) and post-weaning (50 to 95 days of age) periods are given in Table 3. The D1 calves tended to consume higher milk replacer (MR) volumes (*p* = 0.09) compared to CTL calves, and the MR consumption of D2 and D3 calves were not statistically different from CTL calves (*p* = 0.11 and 0.13, respectively). Overall, Sangrovit^®^ seemed to enhance the palatability of MR. The calf starter intake had a cubic relationship with the Sangrovit^®^ dose during the pre-weaning period (*p* < 0.01). The overall relationship across the doses as well as the individual dose effect are useful for understanding the results, so the orthogonal contrast and the mean separations were conducted, respectively. The D1 calves had lower starter intake than that of CTL, D2, and D3 calves (*p* < 0.01), the starter intakes of which were similar to each other (*p* > 0.25). Owing to the higher contribution of the calf starter to DMI relative to the contribution of MR, DMI also had a cubic relationship with the Sangrovit^®^ dose during the pre-weaning period. The dry matter intake of D1 was lower than CTL, D2, and D3 (*p* < 0.01), and DMI of D3 tended to be greater than CTL (*p* = 0.05). Nonetheless, Sangrovit^®^ did not affect ADG or ADG: DMI of pre-weaned calves (Table 3). During post-weaning period, D1 continued to consume less starter compared to CTL (*p* < 0.01), D2 (*p* = 0.04), and D3 (*p* = 0.02). Consequently, the relationship between DMI and the dose of MCE continued to be cubic (*p* = 0.01, Table 3). The relationship between feed intake and the Sangrovit^®^ dose during the pre-weaning and post-weaning period together suggest that the low dose of Sangrovit^®^ could depress calf starter palatability at ingestion as well as post-ingestion levels. However, the absence of such an effect at higher doses is difficult to explain. Despite the changes in DMI, ADG and ADG: DMI remained unaffected by MCE (*p* = 0.68). Previous research [11,17,18,19] also does not show an impact of feeding MCE on the ADG of ruminants. On the other hand, feeding MCE seems to influence individual tissue development and thus the carcass composition of cattle [11]. Therefore, a more detailed assessment in future studies could assist in better understanding the effects of Sangrovit^®^ on the growth and development of calves.

### 3.2. Serum Chemistry

Serum chemistry parameters were evaluated at the end of pre- and post-weaning periods (50 and 95 days of age, respectively) to determine if there is any dose-dependent effect of Sangrovit^®^ on the organs or tissues of calves. Enzymes such as alkaline phosphatase (ALP), alanine aminotransferase (ALT), aspartate aminotransferase (AST), lactate dehydrogenase (LDH), γ-glutamyl transferase (GGT), and creatinine kinase (CK) are sensitive indicators of organ or tissue damage. As shown in Table 4, Sangrovit^®^ dose did not affect serum concentrations of any of those enzymes (*p* > 0.20), except GGT. Overall, the results agree with Toprak et al. [20] showing unchanged serum concentrations of ALP, AST, and ALT in Simmental calves in response to feeding an MCE for 60 d. The GGT concentration decreased with increasing Sangrovit^®^ dose (*p* = 0.04), and the GGT decrease was more prominent post-weaning than pre-weaning (Table 4). Nevertheless, the increasing rather than decreasing plasma GGT concentrations indicate tissue damage. Moreover, a period effect (*p* < 0.01) was observed for all those enzymes except ALP. The concentrations of ALT, AST, LDH, and CK increased, and GGT concentration decreased post-weaning compared to pre-weaning. Those changes are in line with Egli and Blum [21], Knowles et al. [22], Ježek [23], and Mohri et al. [24]. Serum albumin concentration tended to increase linearly with Sangrovit^®^ dose (*p* = 0.06), but the increment (e.g., 3.27 to 3.44 g/dL in suckling calves) would not be of biological significance. Nonetheless, the elevated serum albumin could suggest an improved health status as healthy calves had about 20% greater albumin concentration than calves with chronic respiratory diseases in Tóthová et al. [25]. Sangrovit^®^ dose did affect total protein (TP), and blood urea nitrogen (BUN) concentrations but there was a period effect on BUN (*p* < 0.01). The pre-weaned calves had a lower concentration of BUN in blood compared to weaned calves. The lower BUN of suckling calves was most likely due to a low protein intake compared to that of weaned calves (0.003 vs. 0.004 kg/kg of BW).

Serum creatinine concentration had a quadratic relationship (*p* = 0.01) with Sangrovit^®^ dose, and the relationship was distinct during the pre-weaning period (Table 4). Yu et al. [26] established reference intervals for serum chemistry parameters using data from 134 healthy calves at seven weeks of age. The CREAT changed from 0.43 to 1.03 mg/dL with a mean of 0.66 in that study. The present changes of serum CREAT across Sangrovit^®^ doses were within that range and thus might not alarm a health concern. Neither Sangrovit^®^ dose (*p* ≥ 0.20) nor the period affected (*p* = 0.89) the total bilirubin concentration (TBIL). High values of TBIL could indicate liver diseases and anemia [27]. Blood amylase activity usually helps in assessing pancreatic health. For instance, elevated serum amylase activity indicates acute pancreatitis. Sangrovit^®^ dose did not affect serum amylase activity across pre- and post-weaning periods (*p* ≥ 0.20, Table 4). The reference ranges of serum amylase activity are hard to find in the literature. Animal Health Diagnostic Center at Cornell University established a 14–50 U/L range for healthy cattle. Moreover, Zendehbad et al. [28] demonstrated serum amylase activity above 150 U/L in 2- to 4-month-old Holstein calves with drug-induced mild pancreatitis. Considered together, the present changes in serum amylase activity across Sangrovit^®^ doses do not represent pancreatic damage. Sangrovit^®^ dose did not significantly affect serum concentrations of cholesterol or glucose (*p* ≥ 0.05). The weaned calves had lower serum cholesterol concentrations compared to pre-weaned calves as shown by Carrol et al. [29]. Conversely, weaned calves had higher serum glucose concentrations than pre-weaned calves (*p* < 0.01). The increased serum glucose post-weaning does not agree with the literature [30,31,32]. This discrepancy might partly stem from the fact that the blood of weaned calves was collected in a much colder environment (−10 °C in December) compared to that of suckling calves (10 °C in October) as decreasing air temperatures could increase blood glucose concentrations in ruminants [33]. Calcium (Ca), inorganic phosphorous (iP), sodium (Na), chloride (Cl^-^), and magnesium (Mg) play an important role in maintaining the proper composition of body fluids, and bone, muscle and nerve tissues. Except for potassium, none of the mineral concentrations changed with the administration of Sangrovit^®^ (*p* > 0.05). Potassium concentration decreased linearly (*p* = 0.02) with the Sangrovit^®^ dose. Nevertheless, all mineral concentration measurements were in line with published measurements of healthy calves [21,23,34,35,36,37]. Overall, feeding Sangrovit^®^ did not change the blood chemistry of calves to an extent indicating a health issue or tissue damage.

### 3.3. Hematological Parameters

The hematology parameters measured pre-weaning (~7 weeks of age) and post-weaning (~13 weeks of age) are given in Table 5. Hematology assessment assists in diagnosing blood disorders and organ and tissue defects. The administration of Sangrovit^®^ at any dose did not affect (*p* = 0.52) red blood cell count (RBC), hemoglobin concentration (HGB), or mean corpuscular volume (MCV) in either the pre- or post-weaning periods. Age-related changes were seen in several hematological parameters, as RBC tended (*p* = 0.05) to decrease from pre- to post-weaning periods (10.29 to 9.94 × 10^6^/µL, respectively), whereas HGB concentration (12.12 to 12.96 g/dL) and MCV (34.5 to 36.4 fL) increased (*p* < 0.01) from pre-weaning to post-weaning periods. The RBC, HGB concentration, and MCV measurements were within the ranges of healthy calves at 6 to 16 weeks of age, according to Brun-Hansen et al. [38]. Sangrovit^®^ did also not affect total white blood cell count (WBC) or the counts of sub-populations of WBC (*p* > 0.20, Table 5), except for monocytes. The monocyte count decreased linearly with the Sangrovit^®^ dose (*p* = 0.04). The highest dose (D3) decreased the monocyte counts compared to CTL in weaned calves (0.65 to 0.44 × 10^3^/µL, *p* = 0.02) postulating an improved health state [39]. Nonetheless, all WBC counts in the present study are within the ranges of healthy calves according to Brun-Hansen et al. [38] and Panousis et al. [40].

The platelet count (PLT) had a cubic relationship with the Sangrovit^®^ dose (*p* = 0.03, Table 5). Regardless of the dose, calves receiving Sangrovit^®^ had lower PLT in both pre-weaning (576.7 vs. 728.2 × 10^3^/µL) and post-weaning (533.2 vs. 619.4 × 10^3^/µL) periods (*p* = 0.03). Particularly, D3 tended (*p* = 0.09) to decrease PLT compared to CTL in the post-weaning period (619.4 to 502.2 × 10^3^/µL). Nonetheless, all PLT measurements were within the range of healthy calves [38] and were not viewed as a toxicological action on the calves. Along with platelet counts, fibrinogen concentration, prothrombin time (PT), and activated partial thromboplastin time (APTT) give an insight into the coagulation status of animals. Administration of Sangrovit^®^ to the calves did not change fibrinogen concentration (*p* > 0.30), PT (*p* > 0.80), or APTT (*p* ≥ 0.06). Heuwieser et al. [41] created coagulation profiles for cattle using data from 90 clinically healthy dairy cows and reported fibrinogen concentrations ranging from 125 to 697 mg/dL, PT ranging from 20.1 to 30.1 s, and APTT ranging from 25.3 to 44.5 s. All measurements in the present study fall within the stated reference ranges for calves or cattle [38,39,40,41].

### 3.4. Tissue-Residue Concentrations

Tissue samples (loin, kidney, liver, skin + fat) were retained, packaged individually in plastic specimen bags and analyzed for residual levels of sanguinarine and chelerythrine, Sangrovit^®^ components utilized in past animal studies for tissue-residue analysis [1,7]. The limit of detection (LOD) was confirmed at 0.17 μg/kg and the limit of quantitation (LOQ) was confirmed at 0.5 μg/kg for bovine muscle (loin), liver, kidney, and fat tissues. The samples were analyzed according to an analytical method validated for specificity, linearity, accuracy, precision, range, limit of quantitation, and limit of detection. The results (Table 6) show that increasing levels of consumption of Sangrovit^®^ in the pre-weaning and post-weaning periods generally resulted in increased concentrations of sanguinarine and chelerythrine in the analyzed tissues (e.g., muscle, kidney, liver, and fat).

Fat samples taken from two control animals were found to contain measurable levels of both sanguinarine and chelerythrine. The origin of these resulting levels in the fat samples of two control animals has yet to be determined but was likely due to tissue sample cross-contamination, as no other control samples contained quantifiable levels of sanguinarine or chelerythrine. No other tissues of the control animals that were analyzed contained appreciable or measurable levels (less than 0.5 µg/kg tissue) of sanguinarine or chelerythrine. The limit of quantification validated for this study (less than 0.5 µg/kg tissue) is far lower than previous limits utilized in the Zhao et al. [1] study for these tissues in pigs, which were 45 µg/kg tissue for muscle, fat, and liver, and 60 µg/kg for kidney tissue. Standards and spiked sample recoveries were within acceptance criteria (70–110% recovery for both sanguinarine and chelerythrine). It can be concluded that accurate and reproducible results were obtained utilizing the methodology for this analysis for sanguinarine and chelerythrine in bovine tissues, based on the results obtained from the unknown samples to internal standard calibration curves with points of known values.

## 4. Conclusions

The results of the present study showed no adverse effects of Sangrovit^®^ on the health and growth performance of calves when fed up to 10 g/animal/day during the pre-weaning period (5–49 days of age) and up to 20 g/animal/day post-weaning (50–95 days of age). Sangrovit^®^ increased milk-replacer consumption of suckling calves, but the low dose tended to decrease starter intake in both suckling as well as weaned calves. Sanguinarine and chelerythrine residue levels were located in the muscle, liver, kidney and fat tissues analyzed, with increasing concentrations with increasing Sangrovit^®^ administered, but the levels were considered non-adverse and not a safety concern for the calves. No adverse effects were found in any of the clinical chemistry or hematology parameters evaluated. Overall, no adverse effects were found when suckling and post-weaning calves consumed Sangrovit^®^ at up to 10.0 g/calf/day during the pre-weaning period and 20.0 g/calf/day during the post-weaning period for 90 days.

## Figures and Tables

**Table 1:** Ingredient and nutrient composition of milk replacer and calf starter.

Item	Content
***Milk Replacer***	
**Nutrient composition (% of DM unless otherwise specified) ^1^**	
Crude protein	22.00
Crude fat	20.00
Crude fiber	0.15
Calcium	1.00
Phosphorus	0.70
Vitamin A	9071.85 IU/kg
Vitamin D3	2267.96 IU/kg
Vitamin E	45.36 IU/kg
***Calf Starter***	
**Ingredient composition (% of as-fed) ^1^**	
Whole corn, shelled	54.0
Whole oats	7.5
Heifer pellet ^2^	35.0
Molasses, liquid	2.5
Corn oil	1.0
**Nutrient composition (% of DM, unless otherwise specified) ^3^**	
Dry matter, % as fed	87.9
Crude protein	20.2
Neutral detergent fiber	15.2
Acid detergent fiber	7.3
Starch	41.5
Non-structural carbohydrates	54.0
Crude fat	4.2
Ash	6.3

^1^ Reported by the manufacturer; ^2^ Did not contain antimicrobial, phytogenic, or appetite-enhancing additives and composed of 54.6% soybean meal, 11.7% wheat middlings, 10.0% wheat red dog, 8.4% pork meat and bone meal, 5.3% beet pulp, 4.0% molasses, and 6.0% mineral and vitamin premix (as-fed basis); ^3^ Measured in a certified laboratory; DM = dry matter; IU = International unit; max = maximum; min = minimum.

**Table 2:** The Sangrovit^®^ dose of each experimental treatment.

Treatment Label	Sangrovit Stat Pack^®^ (Pre-Weaning)	Sangrovit G Premix^®^ (Post-Weaning)
CTL	0 g/calf/day	0 g/calf/day
D1	2 g/calf/day	4 g/calf/day
D2	5 g/calf/day	10 g/calf/day
D3	10 g/calf/day	20 g/calf/day

**Table 3:** Effects of Sangrovit^®^ on daily (/d) feed intake, average daily gain (ADG) and feed efficiency (ADG: DMI) during pre-weaning (Sangrovit^®^ Stat Pak) and post-weaning (Sangrovit^®^ G Premix) periods.

Response Variable	Least Squares Means **				SEM ^6^	*p*-Value		
	CTL	D1	D2	D3		Linear	Quadratic	Cubic
Pre-weaning (5–49 d of age) ^1^								
MR intake, L/d	4.90	5.09	5.08	5.06	0.07	0.26	0.17	0.31
Starter intake, kg/d	0.65 ^a^	0.53 ^b^	0.67 ^a^	0.67 ^a^	0.02	<0.01	0.21	<0.01
DMI ^2^, kg/d	1.17 ^a^	1.10 ^b^	1.20 ^a^	1.22 ^a^	0.02	<0.01	0.68	<0.01
ADG, kg/d	0.73	0.69	0.72	0.78	0.06	0.67	0.42	0.65
ADG:DMI	0.62	0.64	0.61	0.64	0.02	0.68	0.64	0.35
BW ^3^, kg	79.70	76.60	77.60	80.00	3.00	0.72	0.44	0.64
Post-weaning (50–95 d of age)								
Starter intake, kg/d	3.42 ^a^	3.20 ^b^	3.38 ^a^	3.28 ^a^	0.05	0.50	0.15	<0.01
DMI ^4^, kg/d	3.05 ^a^	2.85 ^b^	3.01 ^a^	3.01 ^a^	0.04	0.50	0.15	<0.01
ADG, kg/d	1.41	1.35	1.41	1.38	0.11	0.68	0.89	0.75
ADG:DMI	0.45	0.46	0.46	0.45	0.02	0.93	0.88	0.83
BW ^5^, kg	146.10	141.60	145.40	138.90	7.70	0.57	0.86	0.61

^1^ includes the last week of pre-weaning period when milk allowance was reduced from 6.0 to 3.0 kg/d. ^2^ DMI = Dry matter intake; [(milk-replacer intake × 0.12) + (starter intake × 0.88)]. ^3^ body weight at the end of the period (45 d after feeding Sangrovit at approximately 49 d of age). ^4^ DMI = starter intake × 0.88. ^5^ body weight at the end of the study (90 d after feeding Sangrovit and it was around 95 d of age). ^6^ Pooled standard error of the mean (sample size = 10). ** different superscripts represent different (*p* < 0.05) means based on Tukey–Kramer test. CTL = control (0.0 g Sangrovit), D1 = 2.0 g Sangrovit Stat pack pre-weaning and 4.0 g Sangrovit G premix post-weaning, D2 = 5.0 g Sangrovit Stat pack pre-weaning and 10.0 g Sangrovit G premix post-weaning, and D3 = 10.0 g Sangrovit Stat pack pre-weaning and 20.0 g Sangrovit G premix post-weaning; ADG = average daily gain; MR = milk replacer; SEM = Standard error of the mean; Trt = treatment. ^a, b^ different superscripts in a given row indicate different (*p* < 0.05) least squares means.

**Table 4:** The least squares mean and dose-dependent effects of Sangrovit^®^ in the diet on blood-chemistry parameters of pre-weaning and weaned calves.

Parameter	Pre-Weaning (49 d of Age) ^1^				Post-Weaning (95 d of Age) ^2^				SEM ^3^	*p*-Value			
	CTL	D1	D2	D3	CTL	D1	D2	D3		Linear	Quadratic	Cubic	Period
Alkaline phosphatase, U/L	233.70	227.30	214.90	239.90	237.70	227.00	235.80	269.30	21.60	0.30	0.30	0.99	0.37
Alanine aminotransferase, U/L	11.37	11.74	12.05	12.16	15.07	15.24	14.95	15.36	0.89	0.58	0.94	0.85	<0.01
Aspartate aminotransferase, U/L	60.13	55.05	53.42	56.82	74.31	69.65	73.97	79.54	4.02	0.69	0.25	0.50	<0.01
Lactate dehydrogenase, U/L	740.00	721.10	749.40	731.80	837.40	816.10	870.60	846.80	28.5	0.90	0.71	0.22	<0.01
γ-Glutamyltransferase, U/L	18.34	19.53	17.41	15.87	10.04	8.53	9.01	5.77	1.50	0.04	0.63	0.97	<0.01
Creatine kinase, U/L	102.60	85.30	99.40	92.30	138.30	131.40	121.40	136.70	8.5	0.93	0.12	0.58	<0.01
Albumin, g/dL	3.27	3.39	3.33	3.44	3.64	3.79	3.77	3.77	0.06	0.05	0.42	0.10	<0.01
Total protein, g/dL	5.82	6.05	6.05	5.92	5.94	5.98	6.06	5.82	0.12	0.71	0.08	0.80	0.90
Blood urea nitrogen, mg/dL	9.86	11.18	10.63	9.98	11.36	12.87	13.23	12.38	0.85	0.81	0.12	0.42	<0.01
Creatinine, mg/dL	0.84	0.96	0.87	0.95	0.66	0.66	0.67	0.68	0.03	0.07	0.98	0.01	<0.01
Total bilirubin, mg/dL	0.17	0.16	0.11	0.13	0.13	0.17	0.14	0.14	0.03	0.51	0.76	0.20	0.89
Amylase, U/L^3^	13.42	10.89	13.24	13.93	16.26	15.59	17.38	13.45	1.20	0.70	0.68	0.10	<0.01
Cholesterol, mg/dL	73.66	86.70	87.77	85.27	62.16	73.00	69.57	58.47	5.29	0.90	0.05	0.30	<0.01
Glucose, mg/dL	89.31	90.89	93.37	94.87	107.00	103.70	101.90	104.60	3.47	0.65	0.71	0.94	<0.01
Calcium, mg/dL	9.64	9.67	9.82	9.85	10.14	10.27	10.20	10.18	0.15	0.44	0.65	0.92	<0.01
Inorganic phosphorus, mg/dL	8.95	9.48	9.42	9.35	9.64	9.66	9.56	9.65	0.32	0.71	0.65	0.58	0.15
Sodium, mEq/L	139.90	141.00	140.40	141.60	139.10	140.30	139.70	139.50	0.90	0.46	0.75	0.29	0.08
Potassium, mEq/L	4.88	4.94	4.80	4.68	4.80	4.75	4.74	4.47	0.11	0.02	0.49	0.84	0.09
Chloride, mEq/L	101.70	101.40	101.50	101.00	97.40	98.10	98.60	99. 60	0.60	0.34	0.82	0.99	<0.01
Magnesium, mg/dL	2.23	2.24	2.22	2.28	2.21	2.22	2.19	2.29	0.06	0.36	0.52	0.67	0.73

^1^ At the end of the pre-weaning period, calves were approximately 49 days of age and had been receiving Sangrovit^®^ for 45 days. ^2^ At the end of the post-weaning period, calves were approximately 95 days of age and had been receiving Sangrovit^®^ for 90 days. ^3^ Pooled standard error of the mean (sample size = 10). **CTL**= control (0.0 g Sangrovit^®^), **D1** = 2.0 g Sangrovit^®^ Stat pack pre-weaning and 4.0 g Sangrovit^®^ G premix post-weaning, **D2** = 5.0 g Sangrovit^®^ Stat pack pre-weaning and 10.0 g Sangrovit^®^ G premix post-weaning, and **D3** = 10.0 g Sangrovit^®^ Stat pack pre-weaning and 20.0 g Sangrovit^®^ G premix post-weaning.

**Table 5:** The least squares mean and dose-dependent effects of Sangrovit^®^ in the diet on hematological parameters of pre-weaning and weaned calves.

Parameter	Pre-Weaning (49 d of Age) ^1^				Post-Weaning (95 d of Age) ^2^				SEM ^3^	*p*-Value			
	CTL	D1	D2	D3	CTL	D1	D2	D3		Linear	Quadratic	Cubic	Period
WBC, ×10³/µL	8.56	7.55	8.42	8.19	9.18	9.60	8.63	8.37	0.49	0.26	0.84	0.75	0.03
NEUT, ×10³/µL	2.76	2.10	2.75	2.60	2.76	3.08	3.23	2.69	0.29	0.95	0.44	0.23	0.07
LYMPHS, ×10³/µL	4.86	4.87	4.73	4.80	5.47	5.43	5.23	4.96	0.33	0.35	0.93	0.80	0.05
MONO, ×10³/µL	0.67	0.53	0.59	0.57	0.65	0.56	0.59	0.44	0.06	0.04	0.72	0.11	0.46
EOS, ×10³/µL	0.11	0.10	0.14	0.11	0.12	0.11	0.12	0.08	0.02	0.48	0.21	0.25	0.53
BASO, ×10³/µL	0.10	0.09	0.09	0.08	0.17	0.15	0.18	0.16	0.01	0.52	0.93	0.22	<0.01
RBC, ×10^6^/µL	9.98	10.36	10.38	10.45	10.03	9.73	9.76	10.25	0.25	0.91	0.60	0.57	0.05
HGB, g/dL	11.84	12.33	12.14	12.17	12.86	12.94	12.76	13.26	0.27	0.16	0.67	0.87	<0.01
MCH, pg	11.95	11.92	11.65	11.78	12.89	13.15	12.83	12.95	0.17	0.29	0.97	0.27	<0.01
MCH, g/dL	34.43	34.15	34.00	34.23	35.82	35.94	36.01	35.69	0.25	0.54	0.84	0.90	<0.01
MCV, fL	34.76	34.90	34.27	34.41	36.08	36.57	36.25	36.70	0.49	0.98	0.82	0.33	<0.01
PLT, ×10³/µL	728.20	539.50	589.30	601.40	619.40	514.00	583.30	502.20	49.16	0.11	0.20	0.03	0.09
MPV, fL	7.44	7.27	7.54	7.74	6.69	7.46	7.65	7.07	0.29	0.57	0.12	0.49	0.17
Fibrinogen, mg/dL	330.60	299.00	353.10	307.20	268.20	293.60	279.50	260.00	22.47	0.33	0.72	0.48	<0.01
PT, seconds	23.11	22.48	22.22	22.19	24.24	24.41	24.48	24.98	0.40	0.83	0.99	0.92	<0.01
APTT, seconds	30.50	29.95	30.41	28.52	33.50	35.02	31.08	30.58	1.17	0.05	0.31	0.61	<0.01

^1^ At the end of the pre-weaning period, calves were approximately 49 days of age and had been receiving Sangrovit^®^ for 45 days. ^2^ At the end of the post-weaning period, calves were approximately 95 days of age and had been receiving Sangrovit^®^ for 90 days. ^3^ Pooled standard error of the mean (sample size = 10). **CTL**= control (0.0 g Sangrovit^®^), **D1** = 2.0 g Sangrovit^®^ Stat pack pre-weaning and 4.0 g Sangrovit^®^ G premix post-weaning, **D2** = 5.0 g Sangrovit^®^ Stat pack pre-weaning and 10.0 g Sangrovit^®^ G premix post-weaning, and **D3** = 10.0 g Sangrovit^®^ Stat pack pre-weaning and 20.0 g Sangrovit^®^ G premix post-weaning; APTT = Activated partial thromboplastin time; BASO = Absolute basophils; EOS = Absolute eosinophils; HGB = hemoglobin; LYMPHS = Absolute lymphocytes; MCH = Mean corpuscular hemoglobin; MCV = Mean corpuscular volume; MONO = Absolute monocytes; MPV = Mean platelet volume; NEUT = Absolute neutrophils; PLT = Platelet count; PT = Prothrombin time; RBC = Red blood count; WBC = white blood cells.

**Table 6:** Sanguinarine and chelerythrine residue levels (mean ± SD in µg/kg) in tissues of calves slaughtered at 95 d of age after feeding Sangrovit^®^ Stat Pak (5–49 d) and Sangrovit^®^ G Premix (50–95 d).

	CTL	D1	D2	D3
**Sanguinarine (µg/kg)**				
Muscle (loin)	<0.5	1.12 ± 0.45	1.02 ± 0.26	2.16 ± 1.41
Liver	<0.5	3.03 ± 0.924	5.97 ± 1.98	13.3 ± 6.69
Kidney	<0.5	0.55 ± 0.1	0.95 ± 0.26	3.3 ± 1.28
Fat	10.3 ± 1.56	7.8 ± 2.70	10.22 ± 2.09	19.04 ± 4.62
**Chelerythrine (µg/kg)**				
Muscle (loin)	<0.5	0.7 ± 0.14	<0.5	1.83 ± 0.69
Liver	<0.5	0.58 ± 0.05	0.93 ± 0.33	1.91 ± 0.91
Kidney	<0.5	<0.5	<0.5	0.96 ± 0.38
Fat	3.45 ± 0.64	2.13 ± 0.78	2.18 ± 0.76	7.12 ± 2.63

Limit of detection = 0.17 µg/kg tissue. Limit of quantification = 0.5 µg/kg tissue. SD = Standard deviation; CTL = control (0.0 g Sangrovit^®^), D1 = 2.0 g Sangrovit^®^ Stat pack pre-weaning/day and 4.0 g Sangrovit^®^ G premix post-weaning/day, D2 = 5.0 g Sangrovit^®^ Stat pack pre-weaning/day and 10.0 g Sangrovit^®^ G premix post-weaning/day, and D3 = 10.0 g Sangrovit^®^ Stat pack pre-weaning/day and 20.0 g Sangrovit^®^ G premix post-weaning/day.

## Data Availability

All datasets collected and analyzed during the current study are available from the corresponding author on fair request.
